# Feeding difficulties in preterm-born children aged 0–7 years: an observational cohort study

**DOI:** 10.3389/fped.2026.1827238

**Published:** 2026-05-21

**Authors:** Baldassarre Maria Elisabetta, Mileto Vincenzo, Di Mauro Antonio, Cirrottola Serena, Mazzarelli Maria Paola, Graziano Giusi, Pennetta Francesca, Grosso Francesca, Rizzo Valentina, Capozza Manuela, Pellicani Stefano, Laforgia Nicola

**Affiliations:** 1 Department of Interdisciplinary Medicine, Neonatology and NICU Section, University “Aldo Moro”, Bari, Italy; 2 Pediatric Resident, School of Pediatrics, University “Aldo Moro”, Bari, Italy; 3 Pediatric Primary care, National Pediatric Health Care System, ASL BARI, Bari, Italy; 4 Department of Soil, Plant and Food Sciences, University of Bari Aldo Moro, Bari, Italy; 5 Department of Agricultural, Forest and Food Sciences, University of Turin, Turin, Italy; 6 Center for Outcomes Research and Clinical Epidemiology, Pescara, Italy; 7 Unit of Child and Adolescence Neuropsychiatry, Department of Mental Health, ASL Lecce, Lecce, Italy; 8 “Fatebenefratelli” Hospital, Milan, and University “Aldo Moro”, Bari, Italy

**Keywords:** ARFID, feeding difficulties, follow-up studies, pediatric feeding disorder, preterm birth

## Abstract

**Background:**

Preterm-born infants represent a population at high risk for persistent feeding difficulties, yet data on the prevalence of Pediatric Feeding Disorder (PFD) extending through early school age remain limited.

**Methods:**

This observational cohort study enrolled 116 preterm-born children (84 aged 0–3 years; 32 aged 4–7 years) with structured post-NICU follow-up at a single tertiary centre (AOU Policlinico, Bari, Italy), compared with a control group of 33 term-born children. Feeding and relational profiles were assessed using the validated EDQ-C questionnaire (Eating Disorders Questionnaire in Childhood). Associations between perinatal variables and PFD risk were investigated by univariate and multivariate logistic regression.

**Results:**

The prevalence of at least one clinically relevant feeding difficulty was 72.6% in the 0–3-year subgroup and 71.9% in the 4–7-year subgroup. Comparison with term-born controls revealed significantly higher rates of relational disturbance (32.7% vs. 3.0%; *p* = 0.001), food dysregulation (34.6% vs. 12.1%; *p* = 0.021), and selective food refusal (30.8% vs. 12.1%; *p* = 0.048). On multivariate analysis, prolonged nasogastric tube (NGT) use (OR 11.34; 95% CI 1.54–83.60; *p* = 0.017) and lower gestational age (OR 0.56; 95% CI 0.32–0.96; *p* = 0.035) were independently associated with PFD risk. Attachment style was not an independent predictor.

**Conclusion:**

Preterm-born children show substantially higher rates of feeding difficulties compared with term-born peers, persisting through school age. Prolonged NGT exposure and lower gestational age are the primary independent risk factors identified within this structured follow-up cohort, supporting the need for multidisciplinary neurobehavioural surveillance extending beyond the neonatal period.

## 1 Introduction

Nutritional and feeding difficulties in childhood encompass a heterogeneous group of clinical conditions capable of impairing physical and psychosocial development. These conditions are classified within the DSM-5 diagnostic criteria (1–3) and framed internationally within the Pediatric Feeding Disorder (PFD) construct by the consensus definition of Goday et al. (4). The latter identifies PFD as an age-inappropriate impairment of oral intake with dysfunction in at least one of four domains: medical, nutritional, oro-motor skills, and/or psychosocial. The term “feeding difficulties” is used in this study as an operational translation of PFD for the 0–7-year age range, distinct from classical disorders of adolescence and adulthood. The avoidant-restrictive profile that may consolidate at school age is classifiable under DSM-5 as Avoidant/Restrictive Food Intake Disorder (ARFID).

In recent years, neonatal research has placed increasing attention on the impact of preterm birth on the development of these conditions (5). The international literature consistently identifies preterm neonates (gestational age <37 weeks) as a population at high neurodevelopmental risk, with prevalences of nutritional difficulties in the first months of life exceeding 40% (6, 7) and surpassing 60%–70% in cases of severe prematurity enrolled in structured follow-up programmes (8). The underlying vulnerability stems from the complex interaction between neuro-developmental immaturity and neonatal morbidities requiring intensive care management. Among the iatrogenic factors, prolonged enteral feeding via nasogastric tube (NGT) plays a relevant role (9), delaying acquisition of oro-motor skills and promoting oro-pharyngeal sensory aversion (10, 11). These deficits typically manifest in early years as PFD and tend to persist beyond weaning in the absence of targeted interventions, evolving at school age towards an avoidant-restrictive phenotype. The interaction between neonatal clinical parameters and relational variables, such as infant attachment style (12, 13), remains a partially unexplored area of research.

The aim of this study was to assess the prevalence of feeding difficulties in preterm-born patients aged 0–7 years compared with a term-born control group, and to investigate associations with specific clinical and psycho-relational parameters.

## 2 Materials and methods

### 2.1 Study design and participants

This observational cohort study was conducted at the Neonatology and Neonatal Intensive Care Unit of the AOU Policlinico Consorziale, University of Bari “Aldo Moro”, Bari, Italy. A total of 116 preterm-born paediatric patients were enrolled, stratified by chronological age at follow-up into two subgroups: 84 children aged 0–3 years and 32 aged 4–7 years. The control group consisted of 33 term-born children (gestational age ≥37 weeks) recruited at the same centre during the same period. Baseline clinical characteristics of all groups are summarised in Table 1. Parents or legal guardians provided written informed consent for participation and for personal data processing, in accordance with Regulation (EU) 2016/679 (GDPR) and Legislative Decree 196/2003 as amended. Clinical data extracted from the NeoCare management system were processed in anonymised form.

**Table 1:** Baseline clinical characteristics of the study population.

Characteristic	Preterm 0–3 year (*n* = 84)	Preterm 4–7 year (*n* = 32)	Term-born (*n* = 33)	*p*-value
Gestational age (weeks)	32.04 ± 2.79	32.12 ± 3.47	39.42 ± 0.94	**<0** **.** **001**
Birth weight (grams)	1835 ± 650	1887 ± 704	3500 ± 390	**<0** **.** **001**
Male sex, *n* (%)	50 (59.5%)	20 (62.5%)	17 (51.5%)	0.314
Caesarean section, *n* (%)	62 (73.8%)	26 (81.3%)	4 (12.1%)	**<0** **.** **001**
NGT use, *n* (%)	45 (53.6%)	17 (53.1%)	–	–
NGT duratio*n* (days)	22.0 ± 24.9	14.3 ± 22.3	–	–
Parenteral nutrition, *n* (%)	39 (46.4%)	17 (53.1%)	–	–
PN duration (days)	18.3 ± 18.9	9.2 ± 15.1	–	–
Breast milk at discharge, *n* (%)	76 (90.5%)	30 (93.8%)	33 (100%)	**0** **.** **034**
NEC, *n* (%)	1 (1.2%)	0 (0%)	–	–
GI symptoms, *n* (%)	12 (14.3%)	3 (9.4%)	–	–
CNS abnormalities, *n* (%)	2 (2.4%)	1 (3.1%)	–	–
Antibiotic courses, *n* (%)	47 (56.0%)	15 (46.9%)	–	–
PFD, *n* (%)	61 (72.6%)	23 (71.9%)	4 (12.1%)	**<0** **.** **001**

Data are expressed as mean ± SD or *n* (%).

NGT, nasogastric tube; PN, parenteral nutrition; NEC, necrotising enterocolitis; GI, gastrointestinal; CNS, central nervous system; PFD, pediatric feeding disorder; –, not applicable.

The bold values indicate statistical significance.

### 2.2 Data collection and instruments

Clinical and anamnestic data (gestational age, birth weight, mode of delivery, duration and type of enteral/NGT nutritional support, antibiotic courses, neonatal comorbidities) were retrospectively extracted from the NeoCare management system. The behavioural, feeding, and relational profiles of patients were quantified by administering the EDQ-C psychometric questionnaire (Eating Disorders Questionnaire in Childhood) (14) to caregivers, in age-specific versions for the 0–3-year and 4–7-year ranges. A score ≥ 90 on any subscale was considered clinically relevant, according to normative cut-off values.

### 2.3 Statistical analysis

Quantitative variables were described as mean ± standard deviation (SD). The association between clinical variables and PFD onset was assessed using univariate and multivariate logistic regression, calculating Odds Ratios (OR) and 95% Confidence Intervals (95% CI). The number of events per variable (EPV) in the multivariate model was approximately 20, at the lower recommended threshold (15); consequently, point estimates with wide confidence intervals were interpreted conservatively. Comparison between groups was performed using the chi-square test for categorical variables and Student's *t*-test for continuous variables. Statistical significance was set at *p* < 0.05. Analyses were conducted using StataMP17 software.

## 3 Results

### 3.1 Baseline characteristics

Baseline clinical characteristics of the study population are presented in Table 1. The two preterm subgroups were comparable in terms of gestational age (32.04 ± 2.79 vs. 32.12 ± 3.47 weeks) and birth weight (1,835 ± 650 vs. 1,887 ± 704 g). NGT use was similar across the two preterm subgroups (53.6% and 53.1%, respectively). The term-born control group had significantly higher gestational age and birth weight (both *p* < 0.001) and a lower rate of caesarean section (12.1% vs. 73.8%–81.3%; *p* < 0.001).

### 3.2 Prevalence and comparison with term-born controls

The prevalence of at least one clinically relevant feeding difficulty in the preterm group was 72.6% (61/84) in the 0–3-year subgroup and 71.9% (23/32) in the 4–7-year subgroup, compared with 12.1% (4/33) in term-born controls (*p* < 0.001). These figures reflect the specific nature of this cohort, consisting exclusively of patients attending a structured post-NICU follow-up programme at a tertiary referral centre.

Comparison of EDQ-C subscale scores between term-born controls (*n* = 33) and a subsample of 52 preterm infants from the 0–3-year group with complete questionnaire data revealed statistically significant differences in five subscales (Table 2). The largest differences were observed for relational disturbance (32.7% vs. 3.0%; *p* = 0.001), food dysregulation (34.6% vs. 12.1%; *p* = 0.021), and selective food refusal (30.8% vs. 12.1%; *p* = 0.048). Significant differences also emerged for insecure-ambivalent attachment (17.3% vs. 3.0%; *p* = 0.047) and non-emotional anorexia (26.9% vs. 9.1%; *p* = 0.045). No significant differences were found for functional dysphagia, selective eating, rumination, or pica.

**Table 2:** Comparison of EDQ-C subscales (score ≥90) between term-born (*n* = 33) and preterm (*n* = 52; subsample of the 0–3-year group with complete EDQ-C data) children.

EDQ-C Subscale (Score ≥90)	Term-born *n* = 33	Preterm *n* = 52	*p*-value
Relational disturbance	1 (3.0%)	17 (32.7%)	**0** **.** **001** *
Food dysregulation	4 (12.1%)	18 (34.6%)	**0** **.** **021** *
Selective food refusal	4 (12.1%)	16 (30.8%)	**0** **.** **048** *
Insecure-ambivalent attachment	1 (3.0%)	9 (17.3%)	**0** **.** **047** *
Non-emotional anorexia	3 (9.1%)	14 (26.9%)	**0** **.** **045** *
Functional dysphagia	12 (36.4%)	10 (19.2%)	0.079
Insecure-avoidant attachment	11 (33.3%)	8 (15.4%)	0.053
Disorganized attachment	4 (12.1%)	11 (21.2%)	0.287
Selective eating	14 (42.4%)	15 (28.8%)	0.198
Rumination	10 (30.3%)	8 (15.4%)	0.101
Binge eating disorder (BED)	6 (18.2%)	13 (25.0%)	0.462
Bulimia nervosa	6 (18.2%)	10 (19.2%)	0.904
Food phobia	4 (12.1%)	14 (26.9%)	0.104
Affective/mood disorder	3 (9.1%)	7 (13.5%)	0.542
Anxiety disorder	5 (15.2%)	8 (15.4%)	0.977
Conduct disorder	5 (15.2%)	11 (21.2%)	0.490
Loss of control (LOC)	7 (21.2%)	14 (26.9%)	0.552
Pica	2 (6.1%)	5 (9.6%)	0.561

Statistically significant differences are highlighted.

EDQ-C, eating disorders questionnaire in childhood.

* *p* < 0.05.

The bold values indicate statistical significance.

### 3.3 Logistic regression models

Logistic regression analysis (Table 3) identified variables independently associated with PFD development. NGT use emerged as the primary risk factor in both univariate (OR 5.02; 95% CI 1.73–14.60; *p* = 0.003) and multivariate (OR 11.34; 95% CI 1.54–83.60; *p* = 0.017) analyses. The wide confidence interval reflects sample size limitations and warrants caution in interpreting the point estimate.

**Table 3:** Logistic regression analysis for the risk of developing feeding difficulties according to neonatal parameters and attachment style.

Variable	Univariate Model OR (95% CI); *p*-value	Multivariate Model OR (95% CI); *p*-value
Gestational age (weeks)	0.75 (0.59–0.95); *p* = 0.016	0.56 (0.32–0.96); *p* = 0.035
NGT use (Yes vs. No)	5.02 (1.73–14.60); *p* = 0.003	11.34 (1.54–83.60); *p* = 0.017
Birth weight (grams)	0.999 (0.998–1.000); *p* = 0.471 (NS)	1.003 (1.001–1.006); *p* = 0.013
Insecure-avoidant attachment	NS	NS
Insecure-ambivalent attachment	NS	NS
Disorganized attachment	NS	NS

OR, odds ratio; 95% CI, 95% confidence interval; NS, not significant (*p* > 0.05); NGT, nasogastric tube.

Gestational age showed an independent inverse association: each additional week of gestation reduces PFD risk by 44% in the multivariate model (OR 0.56; 95% CI 0.32–0.96; *p* = 0.035). Birth weight was statistically significant only in the multivariate model (OR 1.003; 95% CI 1.001–1.006; *p* = 0.013), being neutral on univariate analysis (OR 0.999; *p* = 0.471), suggesting a probable collinearity artefact with gestational age of no independent clinical relevance. Infant attachment style variables did not reach statistical significance (*p* > 0.05 for all three).

## 4 Discussion

The finding of a PFD prevalence exceeding 72% in preterm-born children enrolled in a structured NICU follow-up programme, stable from early infancy through school age, reaffirms prematurity as a condition of severe neurodevelopmental vulnerability. It should be noted that these prevalence figures pertain specifically to a tertiary referral follow-up cohort and are not intended to be generalised to the broader preterm population, as acknowledged in the Limitations section. The comparison with term-born controls (Table 2) provides a quantitative estimate of differential risk: EDQ-C subscales for relational disturbance, food dysregulation, and selective refusal show 2- to 10-fold higher prevalences in the preterm group.

The persistence of high prevalence in the 4–7-year group supports the evidence that oro-motor challenges arising from neonatal morbidities and their clinical management tend to consolidate into avoidant-restrictive phenotypes (ARFID) at school age, as documented by Park et al. (16) and Brosig et al. (17). In particular, Brosig et al. (17) showed that younger ARFID patients were born preterm significantly more often and had undergone more invasive postnatal procedures, a pattern closely matching that of our cohort. Pados et al. (6) confirmed, in a prospective cohort of infants born before 34 weeks, that problematic feeding difficulties persist at 1, 3, and 6 months of corrected age and are predicted by lower gestational age, consistent with the present findings.

The association found for NGT use (OR 11.34 on multivariate analysis) is biologically consistent with the tube dependency mechanism described by Pahsini et al. (11), mediated by disruption of the hunger-satiety cycle and repeated negative oro-pharyngeal experiences. Alshaikh et al. (9), in a cohort of over 2,100 very preterm neonates, documented that home NGT feeding at discharge is significantly associated with lower gestational age and greater illness severity, confirming the role of NGT as a continuing risk factor beyond NICU admission. We acknowledge, as detailed in the Limitations, that NGT use in this cohort may partly reflect underlying neonatal morbidity severity rather than being an independent causal factor. The wide confidence interval (1.54–83.60), attributable to the limited EPV [∼20; (15)], calls for caution in generalising the point estimate.

The statistically unstable signal for birth weight is most plausibly attributed to collinearity with gestational age and carries negligible independent clinical relevance. This is consistent with findings from Pados et al. (8), whose meta-analysis found that the prevalence of problematic feeding in young preterm-born children was primarily driven by degree of prematurity, with gestational age representing a stronger and more consistent predictor than birth weight alone.

The lack of an independent predictive role for attachment style suggests this variable may operate as a secondary vulnerability factor, amplifying but not causative, in the presence of an organic substrate determined by prematurity and neonatal morbidities. The EDQ-C comparison (Table 2) nonetheless showed a significant difference for insecure-ambivalent attachment (*p* = 0.047), warranting further investigation in adequately powered prospective studies.

### 4.1 Limitations

This study has several limitations that should be considered when interpreting the findings. First, the modest sample size (*n* = 116 preterm; *n* = 33 controls) reduces statistical power and yields estimates with wide confidence intervals. Second, the single tertiary-centre design with structured post-NICU follow-up introduces selection bias: this cohort tends to over-represent children with more complex clinical histories, and the observed prevalence of feeding difficulties should not be generalised to the broader population of preterm-born children not requiring specialised follow-up. Third, neonatal morbidities known to contribute to oral-motor dysfunction and prolonged tube feeding — including bronchopulmonary dysplasia (BPD), intraventricular haemorrhage (IVH), and NEC severity grading — were not systematically recorded as study variables. This limits the ability to fully disentangle the independent contribution of NGT use from underlying illness severity, and represents an important direction for future studies. Fourth, the retrospective design does not allow determination of dose-response relationships for NGT exposure or classification of tube dependency as a distinct clinical entity. Fifth, socioeconomic status and maternal-clinical covariates were not assessed. Sixth, informant bias linked to caregiver traumatic experiences during EDQ-C completion cannot be excluded. Finally, the Italian psychometric validation of the EDQ-C (14) requires further formal documentation in the specific age ranges investigated.

## 5 Conclusion

Within this structured NICU follow-up cohort, preterm-born children showed substantially higher rates of feeding difficulties compared with term-born peers, persisting through early school age. Prolonged NGT exposure and lower gestational age were identified as the primary independent risk factors. These findings support the need for multidisciplinary nutritional and neuropsychological surveillance programmes extending beyond the neonatal period, with follow-up through school age to intercept early transitions towards ARFID phenotypes. Future prospective studies with larger samples, systematic recording of neonatal morbidities, and assessment of socioeconomic covariates are needed to consolidate and extend these findings.

## Data Availability

The raw data supporting the conclusions of this article will be made available by the authors, without undue reservation.
